# Another Dental Patent

**Published:** 1859-07

**Authors:** W. M. Hunter


### ANOTHER DENTAL PATENT.

BY W. M. HUNTER.

IN both the March and June numbers of the *Dental Register*, and in the April number of the *News Letter*, are Editorials, ushering into notice what they call "something new," "a new method of mounting teeth." These notices say *just* enough to excite interest and curiosity, and *too little* to give any information; they were cunningly devised, and their insertion secured by one accustomed to such operations, to serve the special purposes of the so-called inventor of a style of work, which has been experimented with, years ago, and abandoned as impracticable for anything except building-on an additional masticating surface on the inside.

Agents are already canvassing for the sale of instructions and rights, and it was *intended* and *expected* by those most interested, that with the aid of these introductory editorials, with handsome specimens and shrewd agents to travel over the entire country, within a period of six months or a year, that rapid sales for cash could be effected at whatever prices could be obtained, depending entirely on the enthusiasm, gullibility and pecuniary condition of their *victims.* From $40,000 to $50,000, it was thought, would be a small estimate of the amount to be realised from this operation, all of which must be accomplished before the vileness of the humbug could become generally known. Thus it was intended to *fill their pockets* at the pecuniary loss, and, also (unless their eyes were opened before placing work in the mouth,) at the sacrifice of the reputation of those to whom they sold.

A patent has been applied for, and *when issued,* it will be *worth* just precisely its bulk in *blank paper.*

The claim is for "uniting teeth to plate by means of AMALGAM," and forming a rim to the same.

The process is simply this:—Having struck up your plates, ground, arranged and articulated your teeth in the usual way, flatten and bend the pins and invest the outside of teeth and plate in a mixture of plaster and sand or asbestus to retain them in position; then prepare your amalgam of gold or silver (as the case may be) with mercury, (either filings or precipitates may be used,) triturate well and press out as dry as you can with heavy pliars the free mercury; then, with a blunt instrument, pack the amalgam over the teeth down to the plate, to the fullness desired, filling every crevice under the teeth, etc.; then invest the inside of teeth and plate (over the amalgam) with plaster and sand or asbestus, and cut from the outside whatever is necessary to expose the surface to be occupied by the rim; pack on the amalgam as before, and cover with the plaster and sand; then cut two holes, one at each extremity of the jaw, through the investient down to the amalgam, to allow the mercury to escape; then place in the oven of a stove or in any position where a heat of from 680 to 700 degrees can be applied, for four or five hours, or sufficient time to evaporate all the mercury; after the mercury is all evaporated, renew the investients, and if additional strength is required, cover the surface with a solution of borax, and flow solder over it, so that it will pass through the pores or cells down to the plate; then burnish and polish.

It is said that this work is got up "without heat or any soldering whatever." Now, it is apparent that it can not be got up without *heat.* It would be well to examine the *specimens* exhibited, to see if the teeth are not first *backed* and *soldered* on in the usual way. I challenge the authors of this new humbug to produce a piece of work suitable for the mouth, with no other than amalgam union, which I can not break with my thumbs and fingers. Any work which can be so broken, is not sufficiently strong for the mouth, except in *very* favorable cases.

There are a great many reasons why this work is objectionable. I will mention only a few:

1. It is porous, admitting the fluids of the mouth to be absorbed and lodged in the cells vacated by the mercury, from which it can not be removed by any ordinary cleaning; it must, therefore, remain and become putrid and offensive, presenting a beautiful appearance of cleanliness, but in reality, filthy beyond description.

2. Sufficient strength can not be obtained, except by either first backing and soldering in the usual way, or else afterward flowing solder through the cells in the amalgam.

3. The ease, facility and economy with which work can be made, possessing the merits claimed for this, without its faults, viz:

Attaching teeth to plates by the use of block tin, either by pouring like cheoplasty, or by soldering with a tinman's soldering iron; the tin gives sufficient strength, and is free from porosity, therefore clean, and is less subject to oxydation in the mouth than even *pure* silver.

As before remarked, this subject was experimented with, years ago. The difference is simply that those who then experimented had sense enough to stop when they found it *worthless*; but it was left for the genius of a Slayton (inventor of *Slayton's gutta percha base*),—fresh in the memory of the profession—to develope this new and wonderful work, after it had been discarded for years by others. Verily—

"A *little* learning is a dangerous thing."

